# Effects of Elevated CO_2_ on Leaf Senescence, Leaf Nitrogen Resorption, and Late-Season Photosynthesis in *Tilia americana* L.

**DOI:** 10.3389/fpls.2019.01217

**Published:** 2019-10-18

**Authors:** Li Li, Xiaoke Wang, William J. Manning

**Affiliations:** ^1^State Key Laboratory of Urban and Regional Ecology, Research Center for Eco-Environmental Sciences, Chinese Academy of Sciences, Beijing, China; ^2^Co-Innovation Center for Sustainable Forestry in Southern China, Nanjing Forestry University, Nanjing, China; ^3^Bamboo Research Institute, Nanjing Forestry University, Nanjing, China; ^4^Stockbridge School of Agriculture, University of Massachusetts, Amherst, MA, United States

**Keywords:** elevated CO_2_, autumnal leaf falling, late-season photosynthesis, nitrogen resorption, autumnal leaf phenology, *Tilia americana* L

## Abstract

We investigated the effects of elevated CO_2_ concentrations ([CO_2_]) on autumnal leaf falling, late-season photosynthesis, and leaf N resorption efficiency by exposing *Tilia americana* L. to three CO_2_ levels (800 ppm A8, 600 ppm A6, and ambient air A4) in nine continuous stirred tank reactors (CSTRs). All leaves were subdivided into the first (Z1), second (Z2), and third bud break (Z3) leaves. Whole plant photosynthesis (P_Nsat_) was determined by summing the products of P_Nsat_ and total leaf area in Z1, Z2, and Z3, respectively. The results indicated that 1) the timing of leaf senescence in A8 treatments was 21 days in advance, while the senescence duration sustained 20 days longer than ambient treatment; 2) elevated [CO_2_] significantly induced the early formation of overwintering buds, with the number increased by 42 and 29% in A8 and A6 treatments, respectively; 3) Z3 leaf photosynthesis increases consistently until the end of the growing season, but Z2 leaves and whole plant showed acclimation when senescence happened; and 4) autumnal N resorption efficiency in A8 and A6 leaves were 25.5% and 22.7% higher than A4, respectively. In conclusion, autumnal senescence of *T. americana* was accelerated, while the leaf falling duration was extended by elevated [CO_2_]. The change in leaf phenology makes higher N resorption efficiency and earlier and more winter bud formation possible. Meanwhile, a different response of P_Nsat_ within different bud break leaves leads to the disparity between instantaneous measurements of leaf photosynthesis and whole plant photosynthesis in end season.

## Introduction

Carbon dioxide (CO_2_) is essential for the photosynthesis of plants, and future elevated levels of CO_2_ concentrations have been predicted to increase (NOAA, 2019). Effects of elevated levels of CO_2_ on increasing photosynthesis, growth, and biomass of plants have been well documented as a fertilizing effect (Ainsworth and Long, 2005). However, elevated atmospheric CO_2_ concentrations not only stimulate growth of woody species but also alter the growth rhythm of forest trees by modifying the growth cessation timing such as spring bud break (Jach and Ceulemans, 1999), flowering (Krishna et al., 2016), and autumnal leaf senescence (Asshoff et al., 2006; Cleland et al., 2007; Rosenzweig et al., 2008; Norby et al., 2010; Overdieck, 2016). Varied timing of autumnal leaf senescence would have a significant impact on ecosystem productivity. For example, in a deciduous forest of New England, later 10-day canopy senescence in 1994 and 1995 brought about an increase of 500 kg C ha^−1^gross production (Goulden et al., 1996). From previous experiments which were performed with a great range of methods ranging from controlled growth chambers indoor to open-top chambers and free-air CO_2_ enrichment experiment in the field, the results indicated that there had been large variability of forest trees into the autumnal phenophase in response to elevated CO_2_, with advances (e.g., Sigurdsson, 2001; Asshoff et al., 2006; Mcconnaughay et al., 2010; Warren et al., 2011), delays (e.g., Asshoff et al., 2006; Rae et al., 2006; Taylor et al., 2008), or no effect (e.g., Jach and Ceulemans, 1999; Herrick and Thomas, 2003; Norby et al., 2003a, Norby et al., 2003b; Asshoff et al., 2006). Although sink capability (Ainsworth et al., 2004) and nitrogen availability (Sigurdsson, 2001) were proposed as the possible explanations, great uncertainty for the tree autumnal phenology response to elevated CO_2_ still exists.

Leaf photosynthesis is the key aspect of carbon assimilation. The stimulation effects of CO_2_ on leaf net photosynthesis and growth of trees have been fairly well understood (Ainsworth and Long, 2005; Ellsworth et al., 2012; Battipaglia et al., 2013), but most of these measurements were taken once during the middle of the growing season using upper wide-opened leaves. It has been proposed that due to the limited inability of “carbon demand” under CO_2_ enrichment, some species more inclined to early senescence in autumn which will be accompanied with leaf net photosynthesis are decreasing (Keenan et al., 2013). Thus, since the effects of elevated CO_2_ on autumnal phenology may change the relationship between instantaneous measurements of leaf photosynthesis and the annual carbon uptake of a canopy, the net photosynthesis cannot be determined by once instantaneous mid-season measurement without considering leaf age, leaf longevity, and length of the growing season. Less is known about whether changes in elevated CO_2_ may have effects on leaf net photosynthesis in early and late bud break leaves during the late season. The results may also explain some of the inconsistent results between leaf-level photosynthesis and annual growth.

Elevated CO_2_ causes an increase in carbohydrate production through the stimulation of photosynthesis. Nitrogen resorption is defined as the process by which nitrogen is mobilized from senescence leaves and transported to other plant parts such as green leaves, buds, branch, or root (Killingbeck, 1996). When elevated CO_2_ change the life span of leaves, the demand for nutrients from growing or permanent tissues (root, branch) may drive nutrient resorption from senescence of older leaves with lower cost (Kikuzawa and Lechowicz, 2011). In response to atmospheric CO_2_ enrichment, N resorption may become an increasingly important N source as soil nutrients become depleted under rapidly growing vegetation. Because litter N inherently is more difficult to detect and factors that affect senescence and resorption are increasingly variable, few significant effects have been reported on N in senescence leaves and N resorption efficiency response to elevated CO_2_ independently of warming, lacking light, soil nitrogen, and water conditions.

*T. americana* was selected for this experiment because it is a native widespread deciduous tree of North America (Hardin, 1990; Mccarthy, 2012), and the response of *T. americana* to elevated CO_2_ is not well known. The main objective of this experiment is to investigate the timing of autumnal leaf senescence, leaf nitrogen resorption, and late season photosynthesis response to elevated CO_2_ concentration independently of warming, lacking light, soil nitrogen, and water conditions. We wonder, in an enriched CO_2_ environment, whether the growing season in autumn is prolonged combined with sustained high photosynthesis rates and whether more N in senescent leaves is absorbed by trees in the possible lengthened autumn. We hypothesized that (1) elevated CO_2_ concentrations will delay autumn leaf senescence due to the CO_2_ fertilization effect, (2) higher photosynthesis will also sustain with the prolonged growing season in autumn, and (3) more N in senescence leaves will be absorbed back for more N demand of trees, which would also make more overwintering buds formation possible.

## Materials and Methods

### Experimental System and Design

The experiment was carried out in the Laboratory of Plant Environmental Biology in a glass greenhouse at the University of Massachusetts, Amherst, with nine continuously stirred tank reactor (CSTR) chambers. The duration of this experiment was from June 20th to November 28th, 2014 (162 days). The information of CSTRs and CO_2_ control system have been described previously in details (Manning and Krupa, 1992; Elagöz et al., 2006; Albertine and Manning 2009, Li et al., 2018). The temperature inside the greenhouse was averagely 4.7°C higher, while relative humidity was 15% lower than outside, respectively (see Li et al., 2018). The average temperature and relative humidity in 800 and 600 ppm chambers were 0.34°C higher and 2% lower than the control chamber (see Li et al., 2018). CO_2_ concentration enrichment was started from June 20th, 2014 with pure CO_2_ administered continuously for 24 h. The CO_2_ concentration was monitored by one LI-7000 CO_2_/H_2_O analyzers (Li-Cor Inc., Lincoln NE, USA). The LI-7000 CO_2_/H_2_O analyzer was justified every other day, and real-time CO_2_ concentration inside every chamber was checked everyday to ensure that the target CO_2_ concentrations can be reached. Three CO_2_ concentration treatments (800 μl L^–1^ CO_2_ A8, 600 μl L^−1^ CO_2_ A6, and 400 μl L^−1^ CO_2_ A4) were assigned among nine chambers randomly with three replications each. Twenty-seven seedlings in uniform height and basal diameter were divided into three groups with three seedlings in each chamber.

### Plant Material and Management

Two-year-old seedling trees of *T. americana* were obtained from a commercial nursery. After grading for uniformity, seedlings were transplanted into pots (bottom diameter, 18 cm; top diameter, 25 cm; and height, 24 cm) in the greenhouse on June 3rd. The growing medium was Metro Mix 200 (Sun Gro Horticulture).

Trees were acclimated to the greenhouse environment from June 3th to June 25th (23 days). Then, all seedlings were moved inside the CSTRs on June 26th. All seedlings selected were well watered every other day and fertilized with a professional soluble fertilizer (16–17–18; Peters Professional; Scotts, OH, USA) (3.9 g L^−1^) every week. A soluble trace element mix (36.9 mg L^−1^) was fertilized one time on August 8th.

### Gas Exchange Measurements

Leaf light-saturated net photosynthetic rate (P_Nsat_) was measured by a portable Li-Cor 6400 photosynthesis system with a 6400-02B LED light source chamber (Li-Cor Inc., Lincoln NE, USA). The system-controlled saturating photon path‐length probability density function at 1,000 μmol (photo) m^–2^ s^–1^ CO_2_ inside the chamber was supplied by a CO_2_ cylinder, and the CO_2_ concentrations inside the leaf chamber were set similar to the CO_2_ treatments. Two fully expanded leaves were selected from the target bud break leaves, and the measurements were repeated three times. All P_Nsat_ measurements were conducted from 09:00 to 11:30 and 14:00 to 15:00 to avoid the “noon-sleep” phenomenon. Whole plant photosynthesis (P_NsatW_) was determined by the sum of the products between leaf-level photosynthetic rates and leaf area in the first, second, and third bud break leaves.

### Leaf Dynamics Measurements

The trees produced leaves continuously from late April until early July after transplanted into the greenhouse. Leaves were separated and marked into three groups according to the bud break date: the first bud break leaves (Z1) were the oldest leaves which formed by overwintering buds at April, the second bud break leaves (Z2) were formed in the current year after the initial early bud break (approximately at June 3–20), and the third bud break leaves (Z3) were the latest leaves in the current year (early July until 10th). Leaf numbers were counted once every 2 weeks before November, when leaf senescence beginning on October 7th; the frequency was altered to once every 2 days until total abscission. We consider natural autumnal leaf senescence by significant declines in photosynthetic capacity, with the color of the leaves also changing during senescence (Kikuzawa and Lechowicz, 2011). Because there was extremely low temperature or wind in the greenhouse, some senescent leaves without photosynthetic activity remained attached to the stem; these leaves were examined whether to be falling by very gentle shaking. Leaf area was determined by proportional weights, using A4 paper for the leaf prints.

(1) S=k×W

where S is the leaf area (cm^2^), k is the ratio of the area to the weight of A4 paper, and W is the weight of the proportional A4 paper which has the same area as the target *T. americana* leaf.

Whole plant leaf area was calculated by the sum of total leaf area in Z1, Z2, and Z3 leaves.

(2) $$\mathrm{WS}=\sum_{Z1-Z3}S\times \mathrm{Num}$$

where WS is estimated whole plant leaf area, S is the leaf area (cm^2^), and Num is the leaf number. Percentage of abscised leaf area was calculated as follows:

(3) %ALA=(Nabs×S)/WS

where N_abs_ is the abscised leaf number.

### Leaf Mass Per Area and Elemental Carbon and Nitrogen Contents

Leaf mass per unit area (LMA) was determined by measuring mixed 20 disks of leaf dry mass of a known area from five to eight fully expanded leaves on September 15th. Mixed samples of early, middle, and late leaves for elemental contents were collected until the leaf senescence. Elemental C and N contents were measured by an NC2500 elemental analyzer (CE Instruments, Milan, Italy). Nutrient resorption is defined as the process by which nutrients are mobilized from senescence leaves and transported to other plant parts (Killingbeck, 1986). Nitrogen resorption efficiency was calculated as N resorption divided by the initial N content (N in green leaves) as follows (Killingbeck, 1996; Herrick and Thomas, 2003):

(4) $$NRE=100\%\times (N_{g}-N_{s})/N_{g}$$

where N_g_ is the nitrogen contents in green leaves, and N_s_ is the nitrogen in senescent leaves. N_g_ was calculated by the correlation equations between SPAD (a surrogate expressed for chlorophyll content, estimates the amount of chlorophyll present by measuring the amount of light transmitting through a leaf) and N in senescence leaves (Y_N_ = 0.0658X_SPAD_ + 1.5849, R^2^ = 0.67, *P* < 0.05).

### Data Analysis

The parameters of gas exchange and whole leaf area were analyzed by the general multivariate linear model (repeated measure) with elevated CO2 and leaf age (if have) as independent factors (between-subjects variables) and the measured time as a dependent factor (within-subjects variables). For the other parameters except those mentioned above, the CO2 effects were checked by one-way ANOVA and the chamber as an experimental replication unit (three chambers at each CO2 concentration). Post-hoc comparisons were performed with Bonferroni test when the CO2 effect was significant. Results were taken as significant at P < 0.05. Before the analysis, data were checked for normality (Kolmogorov–Smirnov test) and homogeneity of variance (Levene’s test). All data analyses were operated in SPSS statistics software (Version 18.0, SPSS Inc., Chicago, IL, USA).

## Results

### Leaf Demography

**Figure 1:**
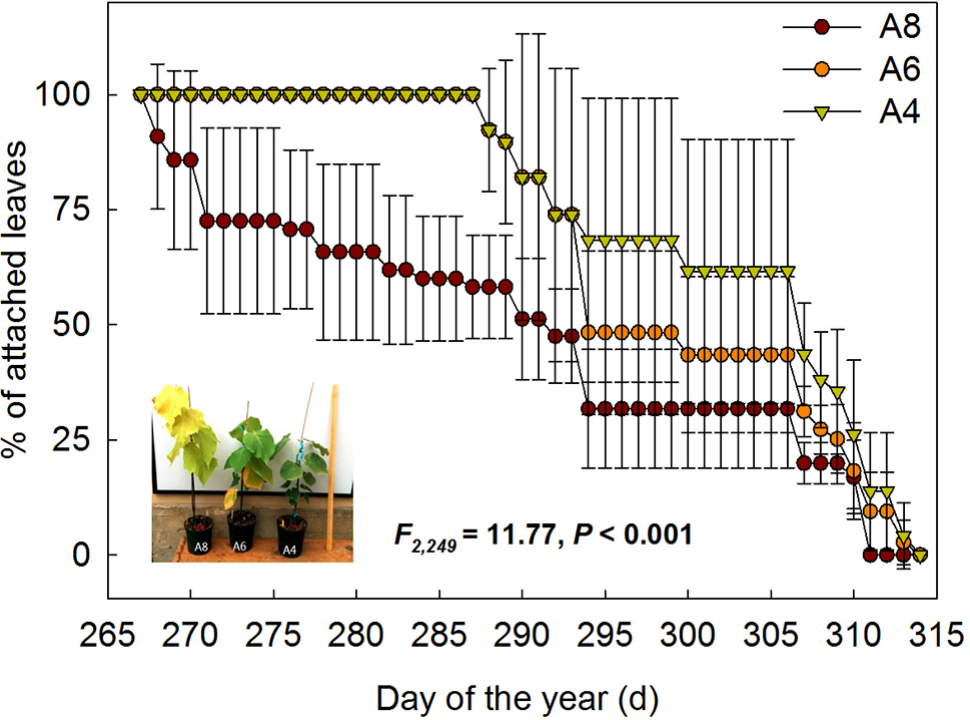
The percentages of attached leaf number of *T. americana* under three CO_2_ concentrations (800 ppm [CO_2_] A8, 600 ppm [CO_2_] A6, and 400 ppm [CO_2_] A4) since autumnal leaf senescence started. Data are represented as mean ± SD. The photo in the graph was taken on September 25th 2014.

To investigate differences in timing of abscission of *T. americana* among three CO_2_ treatments, we plotted the percentages of attached leaves from the start of senescence to the end during autumn (**Figure 1**). The results indicated that the percentages of attached leaf were significantly affected by elevated [CO_2_] (**Figure 1**). After 162 days of CO_2_ fumigation, leaf abscission in A8 started 21 days earlier than A6 and A4, respectively (**Table 1**). No significant effects of elevated CO_2_ on the ending time of leaf abscission were detected (**Table 1**), but the resulting of leaf fall duration in A8 was 20 days longer than A6 and A4 (**Figure 1**; **Table 1**).

**Table 1:** Comparisons of leaf phenology (LP) of the start (SLF) and end time of autumnal leaf falling (ELF), and resulting leaf fall duration (LD) of *T. americana* among three CO_2_ concentration treatments (800 ppm [CO_2_] A8, 600 ppm [CO_2_] A6, and 400 ppm [CO_2_] A4).

LP	A8	A6	A4	P	F_2,6_
SLF	260 ± 2 b	291 ± 3 a	291 ± 9 a	0.04	15.61
ELF	311 ± 0 a	313 ± 3 a	313 ± 3 a	0.34	1.29
LD	42 ± 2 a	22 ± 1 b	22 ± 7 b	<0.01	25.04

Time in the table is the day of the year; each value is mean ± SD. Different lowercase letters after values mean significant multiple comparison results among treatments when P < 0.05.

### Leaf Traits

Elevated [CO_2_] had significant effects on the number of side branches, number of overwinter buds, individual leaf dry weight, and individual leaf area but had no effects on leaf numbers and leaf mass per area (**Table 2**). Individual leaf dry weight and individual leaf area of *T. americana* in A8 were significantly increased by 23 and 33%, and no significant differences were found between A8 and A6 (**Table 2**). The number of side branches and number of overwinter buds of A8 were significantly increased by 225 and 42%, while A6 increased by 150 and 29%, respectively (**Table 2**). We also measured the variation in whole leaf area of the first (Z1), second (Z2), and third bud break (Z3) along with gas exchange measurements.

**Table 2:** The growth parameters (GP) including leaf numbers (LN), numbers of side branches (NSB), numbers of overwinter buds (NOB), individual leaf dry weight (ILDW, g), leaf mass per area (LMA, g m^−2^), and individual leaf area (ILA, cm^2^) of *T. americana* among three CO_2_ concentration treatments (800 ppm [CO_2_] A8, 600 ppm [CO_2_] A6, and 400 ppm [CO_2_] A4).

GP	A8	A6	A4	P	F_2,6_
N	15 ± 3.46 a	16 ± 0 a	12 ± 3.61 a	0.29	1.56
NSB	1.3 ± 0.3 a	1.0 ± 0.4 a	0.4 ± 0.3 b	0.04	5.48
NOB	24.7 ± 0.2 a	22.4 ± 1.9 a	17.4 ± 0.7 b	0.02	9.25
ILDW	1.4 ± 0.1 a	1.3 ± 0.2 ab	1.1 ± 0.0 b	0.04	1.76
LMA	62.6 ± 1.4 a	61.6 ± 3.6 a	61.1 ± 0.5 a	0.52	0.73
ILA	221.7 ± 1.4 a	205.9 ± 3.6 ab	167.3 ± 0.5 b	0.05	5.13

Each value in the table is mean ± SD. Different lowercase letters after number value mean significant multiple comparison results among treatments when P ≤ 0.05.

**Figure 2:**
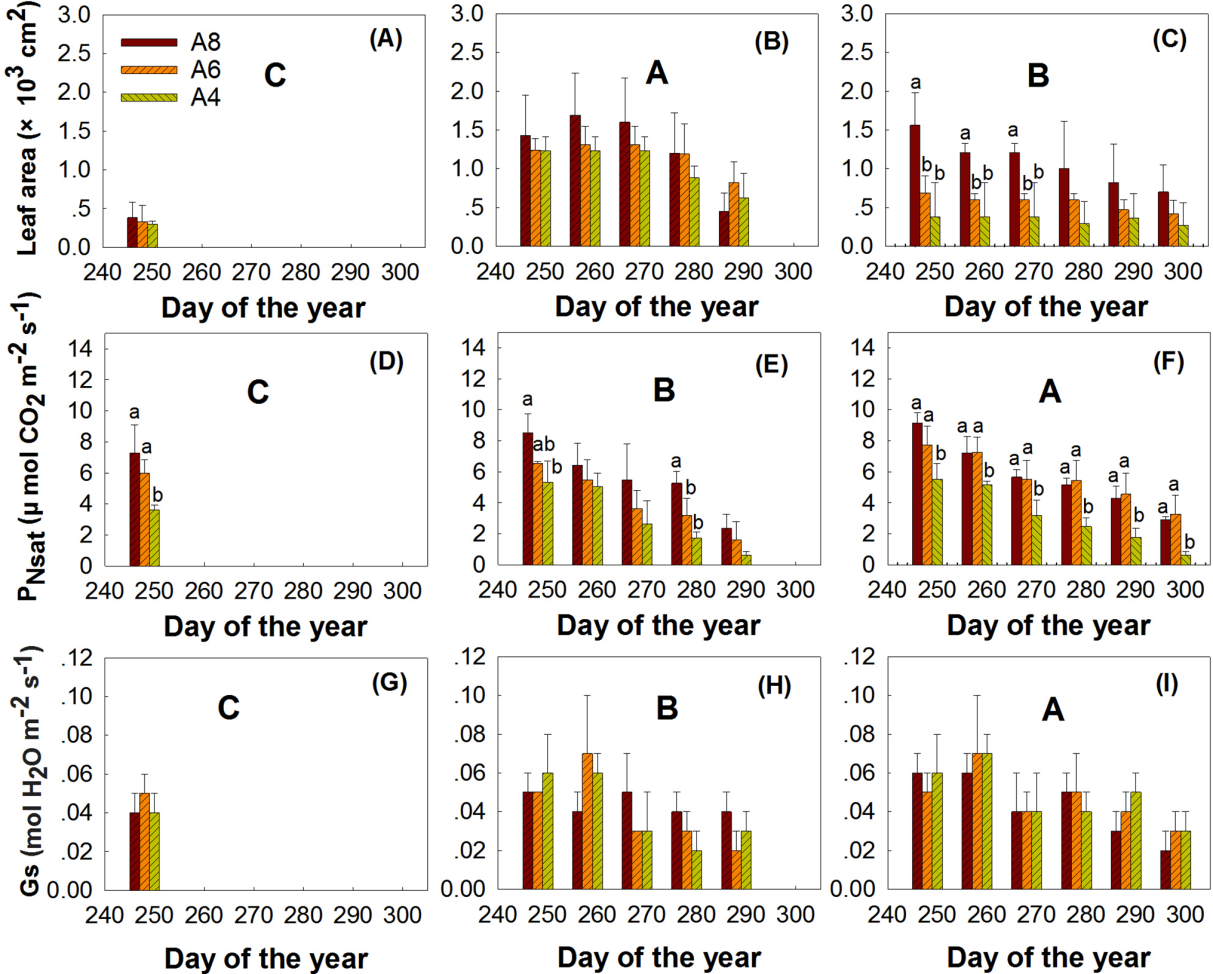
Comparisons of the leaf area, P_Nsat_, and Gs of first budbreak **(A**, **D**, **G)**, second budbreak **(B**, **E**, **H)**, and third bud break **(C**, **F**, **I)** leaves of *T. americana* among three CO_2_ treatments (800 ppm [CO_2_] A8, 600 ppm [CO_2_] A6, and 400 ppm [CO_2_] A4). Data are represented as mean ± SD. Lowercase and uppercase letters mean the multiple comparison results among CO_2_ treatments and measured time when CO_2_ effects are significant at 0.05 level, respectively.

The results indicated that elevated [CO_2_], leaf age, and measured time all significantly affected the whole leaf area at the end of the growing season (**Figure 2**, **Table 3**). Although leaf area significantly declined with time, A8 increased whole leaf area growth and Z2 leaves grew larger than Z1 and Z3. Interaction of measured time and leaf age indicated that whole leaf area of Z2 performed much larger at the beginning of the measurement; the average leaf area of Z2 at the first measured time performed 3.09 and 2.22 times larger than Z1 and Z3 leaves in A4 treatment, respectively (**Table 2**, **Figure 2**).

**Table 3:** ANOVA results of effects of CO_2_ concentration treatments (CO_2_), leaf age (A), measured time (T), and their interactions on leaf area, P_Nsat_, and Gs of *T. americana*.

Factors	CO_2_		A		T		CO_2_ × A		CO_2_ × T		A × T		CO_2_ × A × T	
	*F_2,18_*	*P*	*F_2,18_*	*P*	*F_5,90_*	*P*	*F_4,18_*	*P*	*F_10,90_*	*P*	*F_10,90_*	*P*	*F_20,90_*	*P*
Leaf area	5.31	0.02	40.74	<0.01	51.74	<0.01	2.31	0.10	1.61	0.12	22.85	<0.01	0.71	0.81
P_Nsat_	99.57	<0.01	506.62	<0.01	127.55	<0.01	18.10	<0.01	2.55	0.01	14.76	<0.01	0.89	0.60
Gs	0.36	0.70	279.14	<0.01	30.84	<0.01	1.21	0.34	1.18	0.32	7.79	<0.01	0.89	0.60

### Leaf and Whole Plant Gas Exchange

The results indicated that CO_2_ treatments, leaf age, and measured time all affected P_Nsat_ (**Table 3**, **Figure 2**). A8 and A6 treatments significantly increased P_Nsat_, with P_Nsat_ of Z3 higher than Z2 and Z1 (**Table 3**, **Figure 2**). However, although P_Nsat_ declined with the measured time, the enhancement effects of elevated [CO_2_] on P_Nsat_ of Z3 still sustained until the end of season (**Table 3**, **Figure 2**). A8 and A6 treatments increased P_Nsat_ of Z3 to the highest percentage of 141% and 155%, respectively (**Figure 2**). No significant differences were found between A8 and A6 of P_Nsat_ in Z1 and Z3 (**Table 3**, **Figure 2**). The significant interactions of elevated [CO_2_] and leaf age, elevated [CO_2_] and time, and time and leaf age on P_Nsat_ were detected, which showed that late bud break leaves (Z3), elevated [CO_2_], and earlier measured time would perform higher P_Nsat_ when they combined in pairs.

Stomatal conductance (Gs) was highly variable throughout the senescence period, which was not affected by elevated [CO_2_] but by leaf age, measured time, and the interactions of leaf age and measured time (**Table3**, **Figure 2**). Gs of Z3 leaves was much higher than Z2 and Z1 especially at the beginning of the end season. The highest value of Gs in Z3 appeared at the second measured time, which was 0.07 and 0.01 higher than Gs in Z1 and Z2, respectively.

**Figure 3:**
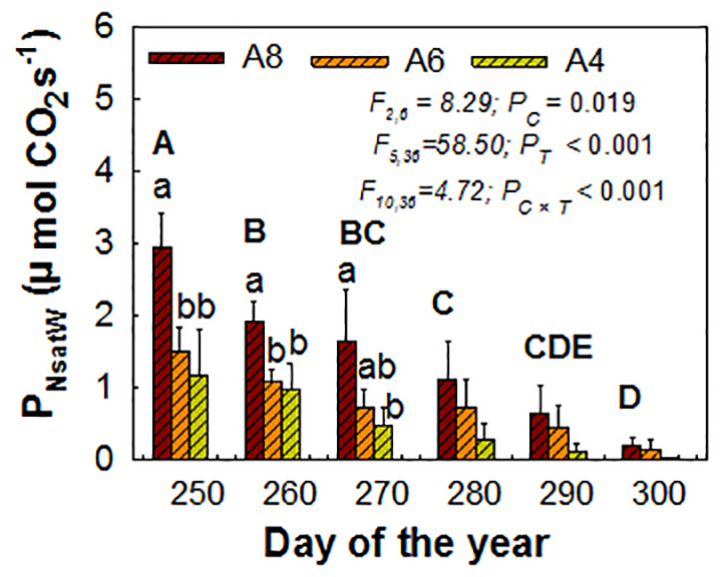
Estimated whole plant P_Nsat_ (P_NsatW_) during the end of the growing season of *T. americana* under three CO_2_ concentrations (800 ppm [CO_2_] A8, 600 ppm [CO_2_] A6, and 400 ppm [CO_2_] A4). Data are represented as mean ± SD. *P*_C_, *P_T_*, and *P_C_*_×_*_T_* mean the *P* values of CO_2_ effects (*P*_C_), time effects (*P_T_*), and their interactions (*P_C_*_×_*_T_*), respectively. Lowercase and uppercase letters mean the multiple comparison results among CO_2_ treatments and measured time when CO_2_ effects are significant at 0.05 level, respectively.

For whole plant P_Nsat_ (P_NsatW_), elevated [CO_2_], measure time, and their interactions were all significant influencing factors. The positive CO_2_ enhancement effects still existed but were not as pronounced until the end, when early senescence happened and was only detected on A8. P_NsatW_ in A8 was increased by 240% to the greatest extent than A4 before senescence happened (the third measured time) (**Figure 3**). A6 had no significant effect on P_NsatW_ at all (**Figure 3**).

### Senescent Leaf Prosperities

**Figure 4:**
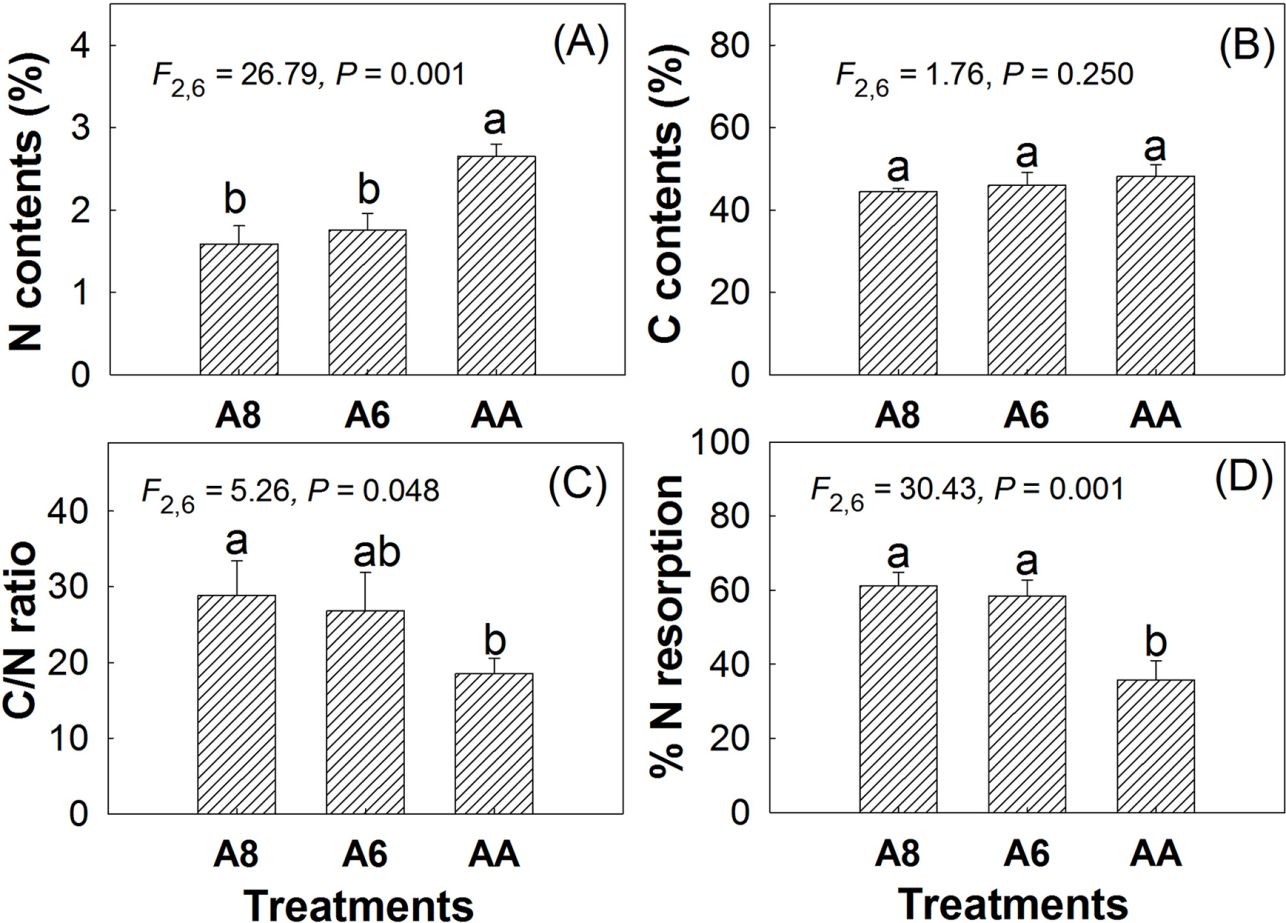
N contents **(A)**, C contents **(B)**, C/N ratio **(C)**, and %N resorption **(D)** of senescent leaves of *T. americana* under three CO_2_ concentrations (800 ppm [CO_2_] A8, 600 ppm [CO_2_] A6, and 400 ppm [CO_2_] A4). Data are represented as mean ± SD. Different lowercase letters above bars mean multiple comparison results among CO_2_ treatments when CO_2_ effects are significant at 0.05 level.

The results of leaf carbon (C) and nitrogen (N) also indicated that elevated CO_2_ had no effects on senescing leaf C contents but significantly decreased leaf N contents. Compared with A4, N contents in A8 and A6 decreased by 40% and 34% individually (**Figure 4**). Thus, the C/N ratio in A8 and A6 was significantly increased by 156% and 145% of A4, individually. No significant differences were found in C, N, and C/N ratio (**Figure 4**) between A8 and A6. N resorption efficiency in senescent leaves of A8 and A6 was significantly higher than that of A4, which were 25.5% and 22.7% higher than A4, respectively.

## Discussion

### Why Leaf Senescence Starts Earlier But With a Longer Duration?

There were few reports on N contents in senescence leaves compared with reports on N decrease in green leaves (Cotrufo et al., 2010). We found that N contents in senescence leaves were decreased by elevated [CO_2_], which means that the lower reduced N contents of green leaves carry over into an increased C/N ratio of senescent leaf litter in autumn (Curtis and Wang, 1998). The possible reasons for N decline include dilution effects (Curtis, 1996), less N demand (sink limitation) (Ainsworth and Long, 2005), less N available (Reich et al., 2006), and CO_2_ inhibition of nitrate assimilation (Bloom et al., 2014).

The significant decline in N contents in senescence leaves is also an indicator of effects of CO_2_ on leaf early senescence (**Figure 4**), which suggests that less nitrogen was invested in photosynthetic light reactions in elevated CO_2_ at the end of the growing season. Senescence, triggered by exogenous or endogenous factors, is an orderly degradation and loss of functions and structures comprising an array of biochemical and physiological processes (Kikuzawa and Lechowicz, 2011). The ultimate goal of senescence is the removal of nutrients from senescing leaves and transport to other plant organ recovery of nutrients from senescing leaves and their recycling within the plant (Lim et al., 2007; Kikuzawa and Lechowicz, 2011). An interesting phenomenon was that, although seedlings in A8 senescence were earlier than in A6 and A4, leaf falling duration in 800 ppm continued 20 days longer (**Table 1**). This phenomenon was related to longer N resorption process for more C fixation, which was due to fertilization effects of the elevated CO_2_ environment. Higher N resorption efficiency in A8 and A6 (**Figure 4**) may make more N transport from senescing leaves to other plant organs possible especially when N is in shortage, no matter due to soil N insufficient or nitrate assimilation inhibition by elevated [CO_2_] (Bloom et al., 2014). Longer senescence duration and higher N resorption efficiency may also be helpful for more overwintering bud formation (**Table 2**) and a possible advanced bud development next year. More overwintering buds were also a sink for C and N transfer, which can be dedicated to early growth in the coming year especially when N availability and uptake is insufficient to meet the demands of the developing foliage (Kang et al., 1982). Leaf longevity is also the result of a trade-off among photosynthetic rates, construction costs, and maintenance costs of a leaf as the leaf tries to maximize whole plant carbon accumulation for a lifetime (Kikuzawa and Lechowicz, 2011), not only for the leaf alone but more generally for the individual plant that bears the leaf (Kikuzawa, 1991).

### Trends in Late-Season Photosynthesis

While most studies took once instantaneous measurements of upper wide-opened leaf photosynthesis in the mid-season, we found the disparity between leaf photosynthesis in different age and canopy photosynthesis during the end season. Leaf photosynthesis in Z3 was stimulated consistently until leaf senescence, while Z2 and whole plant photosynthesis (P_Nsatw_) did not show consistently and pronounced enhancement but acclimation by elevated [CO_2_] (**Figures 2** and **3**; **Table 3**). The high P_Nsat_ in Z3 could be a kind of compensation for early leaf senescence in Z1 and Z2, while whole leaf area decreased, induced by early senescence. The result can be comparable with Takeuchi et al. (2001), who found that downregulation of photosynthetic capacity only showed in the mid and lower canopy, while only the upper canopy leaves were stimulated due to different sink capacity among upper, mid, and lower canopy of *Populus tremuloides* in the Rhinelander free-air CO_2_ enrichment experiment. For the cause for photosynthetic acclimation on Z2, various explanations have been made, including 1) decreased leaf nitrogen (Seneweera et al., 2011; Bloom et al., 2014; Warren et al., 2014), 2) source–sink balance (sink limitations) related to a certain gene (Ainsworth and Long, 2005), and 3) leaf senescence rather than sugar accumulation led to photosynthetic acclimation (Ludewig and Sonnewald, 2000). Apart from this direct effect on photosynthesis, sugar sensing and signaling pathways are reported as important pathways that regulate the photosynthesis process indirectly (Michael et al., 2017). We found N reduction (**Figure 4**), determinate growth (growth date not shown), and leaf early senescence (**Figure 1**, **Table 1**) in the experiment; therefore, this suggests that photosynthetic acclimation in Z2 should be with above multiple processes each contributing to a different degree. However, we are more inclined that the sink limitation reason should take a larger proportion. According to the sink limitation hypothesis, high photosynthetic rates can only be retained while sink capacity (demand for photosynthesis) is high (Ainsworth and Long, 2005). We found that the main stem of *T. americana* stopped to elongate indefinitely due to the determined growth pattern (growth data not shown). Photosynthesis acclimation in elevated CO_2_ is a result of the inability to form sufficient “sinks” for the additional photosynthate (Ainsworth and Long, 2005; Redding et al., 2013), which means that species with a determinate or semideterminate growth are more inclined to have early senescence and downregulation on photosynthesis due to internal reduced “sink demand” for photoassimilation (sink limited) (Herrick and Thomas, 2003; Ainsworth and Long, 2005). It is possible, therefore, that at some point during the experiment, the potential for photosynthetic production exceeded the sink capacity in elevated CO_2_, thereby causing photosynthetic acclimation (downregulation) (**Figure 2**). It happened because the limited medium growth cannot absorb more carbon and nitrogen even in a CO_2_-enriched environment. The differential response of photosynthesis here can also be explained by the cost–benefit theory; no limitations are imposing a longer period of leaf retention, and this is also the optimal timing for leaf turnover at the whole plant level if photosynthetic gains are to be maximized (Kikuzawa and Lechowicz, 2011).

### Shortcomings in the Experiment

We maintained the condition inside the greenhouse in parallel with the real environment outside the greenhouse, e.g., sufficient N and soil water as needed, but due to the seedling size and fumigation period, the impacts of our results were limited in time and space. First, seedlings and saplings in the natural environment must deal with far greater seasonal amplitude of environmental variability than adult trees; 2-year-old seedlings grown from seeds in ambient air possible are more characterized (Huxman et al., 1998; Reich et al., 2006) to elevated CO_2_ than mature, well-established trees for accelerating growth instead of acclimation. Second, the *T. americana* seedlings in the experiment may be still on a sort of priming effect due to the relatively short CO_2_ fumigation period. It was reported that declining N availability (Norby et al., 2010) or downregulation of Rubisco (Rogers and Ellsworth, 2002) happens when some tree species suffered from acclimation of photosynthesis on the long term. Third, although we used bigger pots as the space permitted, the pots possibly restricting the rooting volume should also elicit the sink limitation by limiting belowground sink activity or demand (Mcconnaughay et al., 1993). Therefore, it should be noted that the results occurred when nutrition and water were sufficient, which is a little bit different from most natural environment.

## Conclusions

In summary, a change in either function of leaf phenology or photosynthesis will substantially affect how much carbon the trees gain. We found that elevated CO_2_ accelerates autumnal leaf senescence, but the senescence duration lasted longer. The change in leaf phenology also leads to the disparity between instantaneous measurements of leaf photosynthesis and whole plant photosynthesis during the end season. The early forming of buds may also lead to an early spring bud break the next year, which may also mean a higher risk of frost damage. The higher N resorption efficiency would make more N transported from senescence leaves to other plant parts such as green leaves, buds, branch, or root possibly as an important N source. In an enriched higher CO_2_ in the future, the autumnal phenology performed earlier but longer leaf senescence duration in *T. americana*. The alterations in autumn phenology are essential for predicting how the production of forestry will respond to a future high CO_2_ environment.

## Author Contributions

LL and WM jointly designed the experiment. WM provided financial support. LL ran the experiment, collected and analyzed data, and drafted the manuscript. XW assisted in manuscript revision.

## Funding

This work was funded by the National Natural Science for Youth Foundation of China (31700439) and the National Key Research and Development Program of China (2017YFE0127700), Massachusetts Agricultural Experiment Station.

## Conflict of Interest

The authors declare that the research was conducted in the absence of any commercial or financial relationships that could be construed as a potential conflict of interest.

## References

[B1] Ainsworth E. A. Rogers A. Nelson R. Long S. P. (2004). Testing the “source–sink” hypothesis of down-regulation of photosynthesis in elevated [CO2] in the field with single gene substitutions in *glycine max*. Agric. For. Meteorol. 122 (1), 85–94. 10.1016/j.agrformet.2003.09.002

[B2] Ainsworth E. A. Long S. P. (2005). What have we learned from 15 years of free-air CO2 enrichment (FACE)? A meta-analytic review of the responses of photosynthesis, canopy properties and plant production to rising CO2. New Phytol. 165 (2), 351–372. 10.1111/j.1469-8137.2004.01224.x 15720649

[B3] Albertine J. M. Manning W. J. (2009). Elevated night soil temperatures result in earlier incidence and increased extent of foliar ozone injury to common bean (*Phaseolus vulgaris* L.). Environ. Pollut. 157, 711–713. 10.1016/j.envpol.2008.10.025 19059686

[B4] Asshoff R. Zotz G. Koerner C. (2006). Growth and phenology of mature temperate forest trees in elevated CO2. Glob. Chang. Biol. 12, 848–861. 10.1111/j.1365-2486.2006.01133.x

[B5] Battipaglia G. Saurer M. Cherubini P. Calfapietra C. Mccarthy H. R. Norby R. J. (2013). Elevated CO2 increases tree-level intrinsic water use efficiency: insights from carbon and oxygen isotope analyses in tree rings across three forest face sites. New Phytol. 197 (2), 544–554. 10.1111/nph.12044 23215904

[B6] Bloom A. J. Burger M. Kimball B. A. Jr., and P., P. J. (2014). Nitrate assimilation is inhibited by elevated CO2 in field-grown wheat. Nat. Clim. Chang. 4 (6), 477–480. 10.1038/nclimate2183

[B7] Cleland E. E. Chuine I. Menzel A. Mooney H. A. Schwartz M. D. (2007). Shifting plant phenology in response to global change. Trends Ecol. Evol. 22, 357–365. 10.1016/j.tree.2007.04.003 17478009

[B8] Cotrufo M. F. Ineson P. Scott A. (2010). Elevated CO2 reduces the nitrogen concentration of plant tissues. Glob. Chang. Biol. 4 (1), 43–54. 10.1046/j.1365-2486.1998.00101.x

[B9] Curtis P. S. (1996). A meta-analysis of leaf gas exchange and nitrogen in trees grown under elevated carbon dioxide. Plant Cell Environ. 19 (2), 127–137. 10.1111/j.1365-3040.1996.tb00234.x

[B10] Curtis P. S. Wang X. (1998). A meta-analysis of elevated CO2 effects on woody plant mass, form, and physiology. Oecologia (Berlin) 113 (3), 299–313. 10.1111/j.1365-3040.1996.tb00234.x 28307814

[B11] Elagöz V. Han S. S. Manning W. J. (2006). Acquired changes in stomatal characteristics in response to ozone during plant growth and leaf development of bush beans (*Phaseolus vulgaris* L.) indicate phenotypic plasticity. Environ. Pollut. 140, 395–405. 10.1016/j.envpol.2005.08.024 16202494

[B12] Ellsworth D. S. Thomas R. Crous K. Y. Palmroth S. Ward E. Maier C. (2012). Elevated CO2 affects photosynthetic responses in canopy pine and subcanopy deciduous trees over 10 years: a synthesis from Duke FACE. Glob. Chang. Biol. 18 (1), 223–242. 10.1111/j.1365-2486.2011.02505.x

[B13] Goulden M. L. Munger J. W. Fan S. M. Daube B. C. Wofsy S. C. (1996). Exchange of carbon dioxide by a deciduous forest: response to interannual climate variability. Science 271 (5255), 1576–1578. 10.1126/science.271.5255.1576

[B14] Hardin J. W. (1990). Variation patterns and recognition of varieties of *Tilia americana* s.l. Syst. Bot. 15 (1), 33–48. 10.2307/2419014

[B15] Herrick J. D. Thomas R. B. (2003). Leaf senescence and late-season net photosynthesis of sun and shade leaves of overstory sweetgum (*Liquidambar styraciflua*) grown in elevated and ambient carbon dioxide concentrations. Tree Physiol. 23, 109–118. 10.1093/treephys/23.2.109 12533305

[B16] Huxman T. Hamerlynck E. Jordan D. N. Salsman K. J. Smith S D. (1998). The effects of parental CO2 environment on seed quality and subsequent seedling performance in *Bromusrubens*. Oecologia 114, 202–208. 10.1007/s004420050437 28307933

[B17] Jach M. E. Ceulemans R. (1999). Effects of elevated atmospheric CO2 on phenology, growth and crown structure of Scots pine (*Pinus sylvestris*) seedlings after two years of exposure in the field. Tree Physiol. 19, 289–300. 10.1093/treephys/19.4-5.289 12651572

[B18] Kang S.-M. Ko K. C. Titus J. S. (1982). Mobilization and metabolism of protein and soluble nitrogen during spring growth of apple trees. J. Am. Soc. Hortic. Sci. 107, 209–213.

[B19] Keenan T. F. Hollinger D. Y. Bohrer G. Dragoni D. Munger J. W. Schmid H. P. (2013). Increase in forest water-use efficiency as atmospheric carbon dioxide concentrations rise. Nature 499 (7458), 324. 10.1038/nature12291 23842499

[B20] Killingbeck K. T. (1986). Litterfall dynamics and element use efficiency in a Kansas gallery forest. Am. Midl. Nat. 116 (1), 180–189. 10.2307/2425950

[B21] Killingbeck K. T. (1996). Nutrients in senesced leaves: keys to the search for potential resorption and resorption proficiency. Ecology 77, 1716–1727. 10.2307/2265777

[B22] Kikuzawa K. (1991). A cost–benefit analysis of leaf habit and leaf longevity of trees and their geographical pattern. Am. Nat. 138 (5), 1250–1263. 10.1086/285281

[B23] Kikuzawa K. Lechowicz M. J. (2011). “Ecology of leaf longevity,” in Ecological research monographs, 1st ed (Tokyo: Springer). 10.1007/978-4-431-53918-6

[B24] Krishna J. S. V. Bahuguna R. N. Maduraimuthu D. Rico G. Vara P. P. V. Craufurd P. Q. (2016). Implications of high temperature and elevated CO2 on flowering time in plants. Front. Plant Sci. 7, 913. 10.3389/fpls.2016.00913 27446143 PMC4921480

[B25] Li L. Manning W. J. Wang X. K. (2018). Autumnal leaf abscission of sugar maple is not delayed by atmospheric CO2 enrichment. Photosynthetica 56, 1134. 10.1007/s11099-018-0802-z

[B26] Lim P. O. Kim H. J. Nam H. (2007). Leaf senescence. Annu. Rev. Plant Biol. 58 (1), 115–136. 10.1146/annurev.arplant.57.032905.105316 17177638

[B27] Ludewig F. Sonnewald U. (2000). High CO2 mediated downregulation of photosynthetic gene transcripts is caused by accelerated leaf senescence rather than sugar accumulation. FEBS Lett. 479, 19–24. 10.1016/S0014-5793(00)01873-1 10940381

[B28] Manning W. J. Krupa S. V., (1992). “Experimental methodology for studying the effects of ozone on crops and trees,” in Surface level ozone exposures and their effects on vegetation Chelsea. Ed. Lefohn A. S. (MI: Lewis Publishers, Inc.), 93–156.

[B29] Mccarthy D. (2012). Systematics and phylogeography of the genus *Tilia* in North America. PhD Dissertations & Theses. Chicago: University of Illinois. http://hdl.handle.net/10027/9498.

[B30] Mcconnaughay K. D. Berntson G. M. Bazzaz F. A. (1993). Limitations to CO2-induced growth enhancement in pot studies. Oecologia 94 (4), 550–557. 10.1007/BF00566971 28313996

[B31] Mcconnaughay K. D. M. Bassow S. L. Berntson G. M. Bazzaz F. A. (2010). Leaf senescence and decline of end-of-season gas exchange in five temperate deciduous tree species grown in elevated CO2 concentrations. Glob. Chang. Biol. 2 (1), 25–33. 10.1111/j.1365-2486.1996.tb00046.x

[B32] Michael T. Dananjali G. Naoki H. Anke M. Saman S. (2017). Effects of elevated carbon dioxide on photosynthesis and carbon partitioning: a perspective on root sugar sensing and hormonal crosstalk. Front. Physiol. 8, 578. 10.3389/fphys.2017.00578 28848452 PMC5550704

[B33] NOAA (2019). Trends in Atmospheric Carbon Dioxide, Earth System Research Laboratory-Global Monitoring Division (/gmd/), Global Greenhouse Gas Reference Network (/gmd/ccgg/). https://www.esrl.noaa.gov/gmd/ccgg/trends/ (accessed Sept 10, 2019).

[B34] Norby R. J. Sholtis J. D. Gunderson C. A. Jawdy S. S. (2003a). Leaf dynamics of a deciduous forest canopy: no response to elevated CO2. Oecologia 136 (4), 574–584. 10.1007/s00442-003-1296-2 12811536

[B35] Norby R. J. Hartz-Rubin J. S. Verbrugge M. J. (2003b). Phenological responses in maple to experimental atmospheric warming and CO2 enrichment. Glob. Chang. Biol. 9, 1792–1801. 10.1111/j.1365-2486.2003.00714.x

[B36] Norby R. J. Warren J. M. Iversen C. M. Medlyn B. E. Mcmurtrie R. E. (2010). CO2 enhancement of forest productivity constrained by limited nitrogen availability. PNAS 107 (45), 19368–19373. 10.1073/pnas.1006463107 20974944 PMC2984154

[B37] Overdieck D. (2016). CO2, temperature, and trees. Ecological Research Monographs. Singapore: Springer. 10.1007/978-981-10-1860-2

[B38] Rae A. M. Ferris R. Tallis M. J. Taylor G. (2006). Elucidating genomic regions determining enhanced leaf growth and delayed senescence in elevated CO2. Plant Cell Environ. 29 (9), 1730–1741. 10.1111/j.1365-3040.2006.01545.x 16913862

[B39] Reich P. B. Hobbie S. E. Lee T. Ellsworth D. S. West J. B. Tilman D. (2006). Nitrogen limitation constrains sustainability of ecosystem response to CO2. Nature 440 (7086), 922–925. 10.1038/nature04486 16612381

[B40] Redding E. Fernandez I. Day M. Wiersma G. B. (2013). Phenology at the Bear Brook Watershed in Maine, USA: Foliar Chemistry and Morphology. Am. J. Plant Sci. 04, 14. 10.4236/ajps.2013.412293

[B41] Rogers A. Ellsworth D. S. (2002). Photosynthetic acclimation of *Pinus taeda* (loblolly pine) to long-term growth in elevated CO2. Plant Cell Environ. 25, 851–858. 10.1046/j.1365-3040.2002.00868.x

[B42] Rosenzweig C. Karoly D. Vicarelli M. Neofotis P. Wu Q. Casassa G. (2008). Attributing physical and biological impacts to anthropogenic climate change. Nature 453 (7193), 353–357. 10.1038/nature06937 18480817

[B43] Seneweera S. Makino A. Hirotsu N. Norton R. Suzuki Y. (2011). New insight into photosynthetic acclimation to elevated CO2: the role of leaf nitrogen and ribulose-1,5-bisphosphate carboxylase/oxygenase content in rice leaves. Environ. Exp. Bot. 71, 128–136. 10.1016/j.envexpbot.2010.11.002

[B44] Sigurdsson B. (2001). Elevated CO2 and nutrient status modified leaf phenology and growth rhythm of young *Populus trichocarpa* trees in a 3-year field study. Trees 15, 403–413. 10.1007/s004680100121

[B45] Takeuchi Y. Kubiske M. E. Isebrands J. G. Pregitzer K. S. Hendrey G. Karnosky D F. (2001). Photosynthesis, light and nitrogen relationships in a young deciduous forest canopy under open-air CO2 enrichment. Plant Cell Environ. 24, 1257–1268. 10.1046/j.0016-8025.2001.00787.x

[B46] Taylor G. Tallis M. J. Giardina C. P. Percy K. E. Miglietta F. Gupta P. S. (2008). Future atmospheric CO2 leads to delayed autumnal senescence. Glob. Chang. Biol. 14, 264–275. 10.1111/j.1365-2486.2007.01473.x

[B47] Warren J. M. Norby R. J. Wullschleger S. D. (2011). Elevated CO2 enhances leaf senescence during extreme drought in a temperate forest. Tree Physiol. 31, 117–130. 10.1093/treephys/tpr002 21427157

[B48] Warren J. M. Jensen A. M. Medlyn B. E. Norby R. J. Tissue D. T. (2014). Carbon dioxide stimulation of photosynthesis in *Liquidambar styraciflua* is not sustained during a 12-year field experiment. AoB Plants 7, 074. 10.1093/aobpla/plu074 PMC4294433 25406304

